# Ciprofloxacin Enhances Stress Erythropoiesis in Spleen and Increases Survival after Whole-Body Irradiation Combined with Skin-Wound Trauma

**DOI:** 10.1371/journal.pone.0090448

**Published:** 2014-02-28

**Authors:** Risaku Fukumoto, True M. Burns, Juliann G. Kiang

**Affiliations:** 1 Radiation Combined Injury Program, Armed Forces Radiobiology Research Institute, Bethesda, Maryland, United States of America; 2 Department of Radiation Biology, Uniformed Services University of the Health Sciences, Bethesda, Maryland, United States of America; 3 Department of Medicine, Uniformed Services University of the Health Sciences, Bethesda, Maryland, United States of America; French National Centre for Scientific Research, France

## Abstract

Severe hematopoietic loss is one of the major therapeutic targets after radiation-combined injury (CI), a kind of injury resulting from radiation exposure combined with other traumas. In this study, we tested the use of ciprofloxacin (CIP) as a treatment, because of recently reported immunomodulatory effects against CI that may improve hematopoiesis. The CIP regimen was a daily, oral dose for 3 weeks, with the first dose 2 h after CI. CIP treatment improved 30-day survival in mice at 80% compared to 35% for untreated controls. Study of early changes in hematological parameters identified CI-induced progressive anemia by 10 days that CIP significantly ameliorated. CI induced erythropoietin (EPO) mRNA in kidney and protein in kidney and serum; CIP stimulated EPO mRNA expression. In spleens of CI mice, CIP induced bone morphogenetic protein 4 (BMP4) in macrophages with EPO receptors. Splenocytes from CIP-treated CI mice formed CD71^+^ colony-forming unit-erythroid significantly better than those from controls. Thus, CIP-mediated BMP4-dependent stress erythropoiesis may play a role in improving survival after CI.

## Introduction

Victims of nuclear disasters often suffer from a combination of radiation injury (RI) along with other insults such as physical wounds and thermal burns, a class of injury designated as radiation-combined injury (CI). Development of countermeasures to CI has been a pressing need that requires understanding the synergism between radiation and other insults.

In order to understand the pathology associated with CI, we previously established an experimental model in which mice received radiation alone or radiation followed by wounding. The model demonstrated that a non-lethal wounding administered to irradiated animals consistently reduced 30-day survival [1]. Study of CI animals at early time points revealed enhanced DNA breaks, blood cell depletion, increases in several pro-inflammatory cytokines, tissue damage in several organs, and endogenous bacterial translocation causing lethal septic shock [1]–[3]. These unique changes offer potentially valuable targets for treating CI.

Among the enhanced changes after CI, erythrocyte depletion drew our attention. It has been reported in a rat model that RI and thermal burn have a synergistic effect on anemia development [4]. Anemia is a condition described as a low level of hemoglobin [5]. It causes lack of oxygen delivery, and when it becomes severe, organ failure and fatality can occur [6]. There are several causes known for anemia: rapid loss of preexisting erythrocytes by hemorrhage and hemolysis causes acute anemia, while inflammation induced by infection or autoimmune diseases impairs erythropoiesis and causes anemia of chronic disease (ACD), the most frequent type of anemia treated in hospitals [7].

It is known in mice that “stress erythropoiesis” in the spleen rapidly develops in response to anemia, while “homeostatic erythropoiesis” takes place mainly in bone marrow to maintain healthy erythrocyte levels. The pathway responsible for stress erythropoiesis is initiated by production of erythropoietin (EPO) in the kidney, the organ that senses low oxygen tension. EPO in turn stimulates macrophages present in the spleen to produce bone morphogenetic protein 4 (BMP4), an essential cytokine for expansion and differentiation of immature erythrocyte progenitors to become stress burst-forming unit-erythroid (sBFU-E) [7]. Under inflammation, sBFU-E respond to EPO and differentiate into colony-forming unit-erythroid (CFU-E) and further to mature erythrocytes, while homeostatic BFU-E in bone marrow show little response [7]. The mechanisms underlying anemia after CI have not been studied.

Ciprofloxacin (CIP) is a commonly used fluoroquinolone (FQ), whose clinical application is approved by the U.S. Food and Drug Administration (FDA) for antimicrobial use. In addition to this antimicrobial effect, however, immunomodulatory effects have also been reported in rodent models and human clinical trials that have been shown to improve a wide range of conditions, including inflammatory bowel disease [8], [9], rheumatoid arthritis [10], and chemotherapy-induced neutropenia [11]. These observations convincingly demonstrate that immunomodulation is a result of two general effects of CIP; control of inflammation and stimulation of hematopoiesis.

In our recent report, we showed that CIP reduced massive production of pro-inflammatory cytokines interleukin (IL)-1 and IL-6 while stimulating IL-3 expression in serum of mice 10 days after CI [3]. It was not determined, however, if CIP stimulated stress erythropoiesis and improved overall survival through these immunomodulatory effects.

We are here the first to report that CIP treatment greatly improves 30-day survival in CI mice compared to untreated mice. In our experiments, CI mice developed severe anemia over the course of 10 days that was partially ameliorated by CIP treatment. CIP treatment increased BMP4 production by F4/80^+^ macrophages in spleen, although CIP did not significantly increase EPO during CI. In an *ex vivo* colony formation assay, splenocytes isolated from CIP-treated CI mice consistently gave rise to CFU-E in the presence of EPO and IL-3. Thus, CIP-mediated stimulation of stress erythropoiesis may play an important role in CIP efficacy against CI-induced mortality.

## Results

### Ciprofloxacin Improves Survival after RI and CI

**Figure 1:**
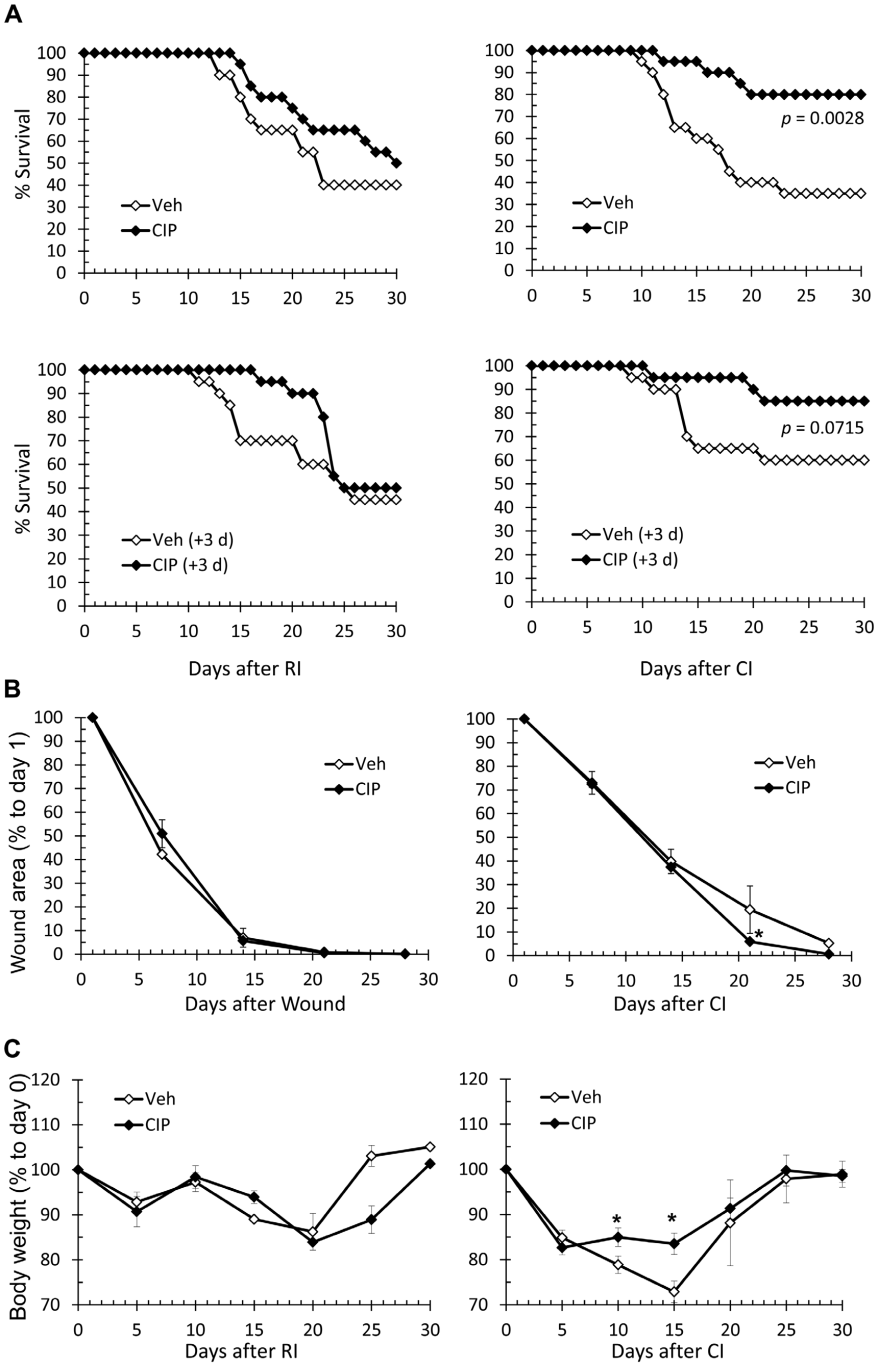
CIP increases survival, accelerates wound closure, and ameliorates body weight loss after CI. Mice received 9.25/or 15% TBSA wounding to establish following four treatment groups: CI, RI, Wound, or Sham. Mice in each group received daily, oral doses of CIP or Veh for 3 weeks starting 2 hours after injury unless otherwise noted. *p<0.05 for the values between groups. (A) Top: Daily survival rates for RI (left) and CI (right) mice treated with CIP or Veh. Bottom: Daily survival rates for RI (left) and CI (right) mice treated with CIP (+3 d) or Veh (+3 d) regimens where CIP treatment was same except initiated as late as 3 days after CI. No mice died in Sham and Wound groups (data not shown). *n* = 20/group. Mantel-Cox test was used to evaluate statistical significance. (B) Average wound closure rates for Wound (left) and CI (right) mice treated with CIP or Veh. Data are presented as percentage of day 1 average wound size. (C) Average body weight rates for RI (left) and CI (right) mice treated with CIP or Veh. Data are presented as percentage of day 0 average body weight. CIP: ciprofloxacin; Veh: vehicle; CI: combined injury; RI: radiation injury; Wound: wounding alone; Sham: free of any injury.

We exposed mice to 9.25 Gy radiation and/or 15% total body surface area (TBSA) wounding to produce the following four treatment groups: radiation+wounding (CI), radiation alone (RI), wounding alone (Wound), and free of any injury (Sham). Mice in each of these groups received CIP or vehicle (Veh) to produce a total of eight groups. The severity of CI produced by these treatments should represent an LD_70/30_ according to previous studies [1]; a mortality of 65% was in fact observed (Figure 1A top right panel). The onsets of mortality were days 13 and 10 in RI and CI mice, respectively (Figure 1A top left panel vs. top right panel). None of mice in the Sham and Wound groups died, indicating 15% TBSA wounding was non-lethal.

We used a CIP regimen of daily, oral doses for 3 weeks starting 2 h after CI. CIP treatment significantly improved CI mice survival compared to that measured in Veh-treated controls. CIP delayed onsets of mortality in both RI and CI mice (days 15 and 12, respectively). Moreover, CIP treatment after CI improved 30-day survival from 35% measured in Veh-treated controls to 80% (Figure 1A top right panel). Mice treated with CIP after RI showed an increase in 30-day survival from 40% to 50%, but the increase was not significant (Figure 1A top left panel). In addition, none of the CI mice died after completion of CIP therapy on day 21, indicating this regimen was effective. Similarly, CIP improved survival in RI mice through 26 days. However, 20% of mortality occurred after completion of CIP therapy (Figure1A, left panel). We tried another regimen (+3 d) where CIP treatment was the same except initiated as late as 3 days after CI; we obtained similar results. The effect of delayed therapy was observable in CI (Figure 1A bottom right panel: 85% vs. 60%), but not in RI mice (Figure 1A bottom left panel: 50% vs. 45%). After completion of CIP therapy on day 21, none of the CI mice had died while 40% of RI mice died (Figure 1A bottom panels).

### Ciprofloxacin Accelerates Wound Healing after CI

It is known that RI slows wound healing [1], [2]. We, therefore, studied the effect of CIP on wound sizes observed weekly after Wound or CI injuries. In sham-irradiated mice, wound sizes (expressed as a percentage of the day 1 average size) decreased at a consistent rate by days 7 and 14 regardless of CIP treatment (Figure 1B, left panel). Complete wound healing was observed by day 21 in all mice. With CI, as previously reported, closing rates significantly slowed compared to groups that received wound alone (Figure 1B, right panel). CIP treatment did not lead to improved wound healing compared to Veh-treated groups by days 7 (72.46% vs. 73.02%) and 14 (37.40% vs. 39.84%); however, significant improvement was observed by days 21 (5.89% vs. 19.43%) and 28 (0.65% vs. 5.25%). CIP (+3 d) treatment also improved wound closures (data not shown).

### Ciprofloxacin Alleviates Body Weight Loss after CI

It has been shown that CI leads to faster body weight loss than does RI [1]. We, therefore, investigated the effect of CIP treatment on body weights after RI and CI. Results are expressed as percentage of the day 0 average. Consistent with the previous report, CI induced a loss in body weight more rapidly and severely than did RI (Figure1C left vs. right panels). Average body weights reached a nadir on day 15 (72.9%) after CI and on day 20 (86.2%) after RI. CIP treatment led to significantly less body-weight reduction after CI (83.5%; Figure 1C, right panel). CIP treatment did not improve body weight after RI (Figure 1C, left panel). The degree of recovery in body weights after their nadirs was related to mortality, which mostly occurred in lower-weight mice. Similarly, CIP (+3 d) treatment improved body weight (data not shown).

### Ciprofloxacin Mitigates Erythrocyte Loss after CI

**Figure 2:**
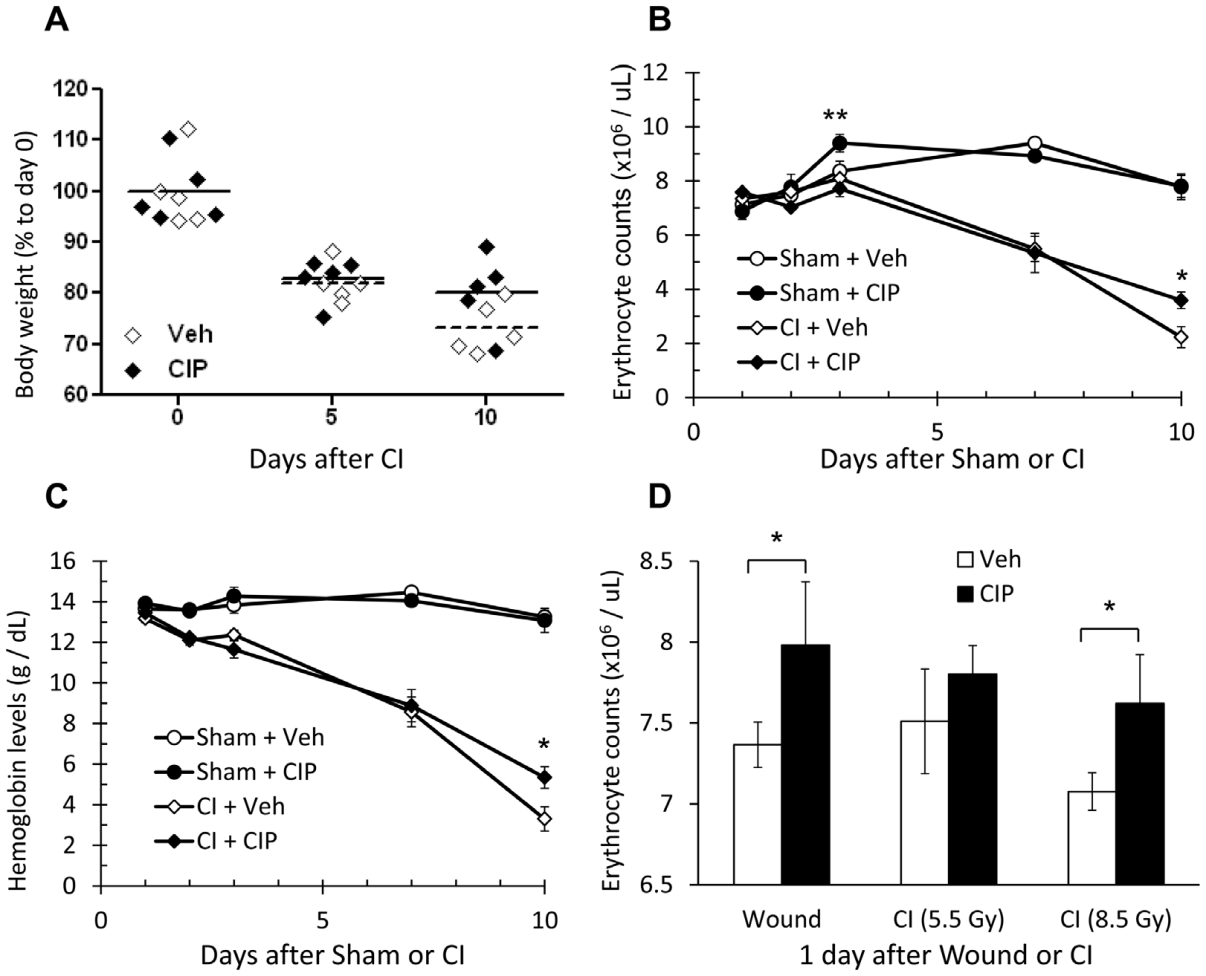
CIP improves anemia induced by CI. Mice received CI and were treated with daily, oral doses of CIP or Veh starting 2 hours after injury. Erythrocytes and hemoglobin levels were analyzed over course of 10 days. (A) Body weight rates for CI mice 5 and 10 days after CI. Individual and average body weights from each group are shown. Data are presented as percentage of day 0 average body weight. (B) Erythrocyte counts at 1, 2, 3, 7 and 10 days after CI. *p<0.05 between CI+Veh and CI+CIP; **p<0.1 between Sham+Veh and Sham+CIP. (C) Hemoglobin levels at 1, 2, 3, 7 and 10 days after CI. *p<0.05 between CI+Veh and CI+CIP. (D) Erythrocyte counts at 1 day after wounding with increasing doses of radiation. *p<0.2. CIP: ciprofloxacin; Veh: vehicle; CI: combined injury; Wound: wounding alone; Sham: free of any injury.

Our survival and body weight studies revealed that CIP dramatically improved the condition of CI but not RI mice. CIP also accelerated wound healing in CI animals. Furthermore, we recently reported that CIP significantly reduces massive release of proinflammatory cytokines, including IL-6, KC and Rantes, while potentiating other cytokines such as IL-3 and GM-CSF by day 10 after CI [3]. These cytokines are known in general to stimulate hematopoiesis, including erythropoiesis; however, in those studies they did not improve total white blood cell or platelet numbers. We, therefore, conducted a separate experiment, in which the effect of CIP on other hematological changes was observed on days 1, 2, 3, 7 and 10 after CI (9.75 Gy, 15% TBSA wounding). Body weight data obtained on days 5 and 10 were similar to that observed in the survival study (Figure 1C right panel vs. 2A). We found a severe, progressive loss in erythrocytes from mice that received CI, resulting in a reduction to 30.37% on day 10 relative to the values measured on day 1 (Figure 2B). Corresponding to this, the levels of hemoglobin decreased to 25.06% (Figure 2C) and hematocrit to 29.27% (data not shown). These values reach the level indicative of anemia. CIP treatment significantly improved erythrocyte counts to 47.31% (Figure 2B), hemoglobin levels to 39.70% (Figure 2C), and hematocrit to 47.20% (data not shown). Interestingly, CIP alone also transiently stimulated the erythrocyte counts in Sham animals, increasing to a statistically significant 136.70% on day 3(Figure 2B). We next investigated the effect of 0, 5.5, or 8.5 Gy radiation combined with a 15% TBSA wound on erythrocyte counts. On day 1 we found a consistent increase in erythrocyte counts in CIP-treated animals regardless of the types of injury. While some of the changes were significant (Figure 2D), significance was lost by day 7 (data not shown).

**Figure 3:**
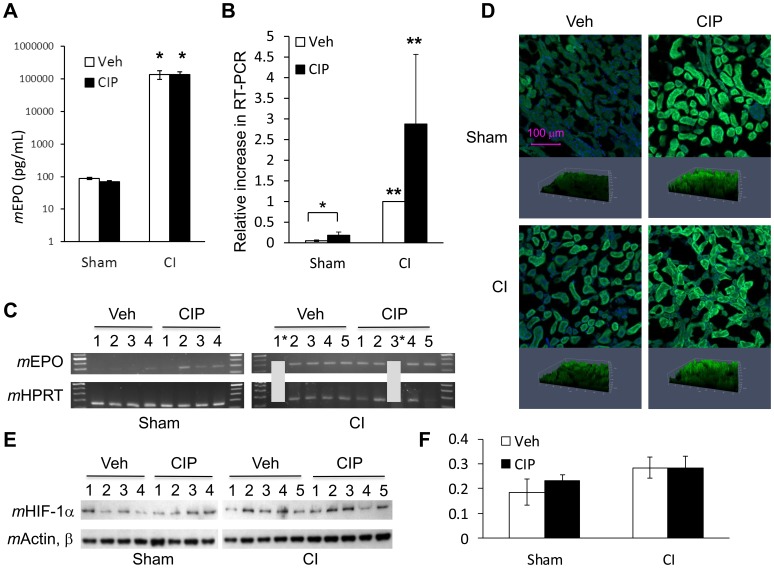
CIP stimulates EPO mRNA expression. Mice received CI or Sham and were treated with daily, oral doses of CIP or Veh starting 2 hours after injury. Levels of EPO in sera, and EPO mRNA and HIF-1α in kidney were analyzed 10 days after CI. (A) Serum EPO levels: ELISA. *p<0.01 compared to others. (B and C) Kidney EPO levels: real-time PCR (B) and actual PCR products at 30 cycle point (C). (B) *p<0.05 and **p<0.01 compared to others; (C) *indicates no amplification was performed due to limited sample yield. (D, E and F) Kidney HIF-1α levels: immunofluorescent staining (D), immunoblots and their quantitations (E and F). Annotation in top-left image in (D) applies to all other images. CIP: ciprofloxacin; Veh: vehicle; CI: combined injury; Sham: free of any injury.

Hematologic analysis indicated that CIP could stimulate erythropoiesis shortly after CI as well as under a severe anemic condition on day 10. Perhaps, CIP stimulated erythrocyte progenitors before they underwent CI-induced apoptosis along with the newly formed progenitors under anemic stress. In anemic conditions, kidneys generally sense low oxygen tension and produce erythropoietin (EPO), an essential hormone for erythropoiesis [12]. Kidneys are also known to be less sensitive to radiation relative to other organs such as ileum or bone marrow [13]. We initially hypothesized that CIP stimulated EPO production and, therefore, tested EPO levels in sera and kidney at day 10 after CI. We found that CI increased serum levels of EPO regardless of the presence or absence of CIP (Figure 3A). Using qRT-PCR analysis, we found CI significantly increased the level of EPO mRNA in kidney; CIP treatment also significantly increased expression in Sham but not in CI mice (Figure 3B and C). Since transcription of EPO gene is mainly regulated by hypoxia-inducible factor 1-alpha (HIF-1α), a transcription factor, we investigated the nuclear translocation of HIF-1α, as well as its protein levels in total cell lysates. Immunofluorescent staining of renal sections demonstrated that significant amounts of HIF-1α are present in nuclei after CI. CIP treatment increased translocation in the absence of CI (Figure 3D). Analysis performed on kidney lysates from the same animals, however, revealed neither CI nor CIP significantly changed the total amount of HIF-1α (Figure 3E and F). CI but not CIP greatly induced EPO protein in kidney lysates (data not shown).

### Ciprofloxacin Increases BMP4 in F4/80^+^ Cells with EPO Receptor

EPO normally stimulates homeostatic erythropoiesis by binding EPO receptors (EpoR) present on erythrocyte progenitors in bone marrow [14], [15]. However, in anemic conditions induced by chronic inflammation or reticulocytopenia, it stimulates “stress erythropoiesis” in the spleen [7]. We reported dramatic loss of bone marrow cells of animals after 10 days of CI, suggesting their poor response to EPO stimulation [3]. It is believed that F4/80^+^ macrophages in the spleen mediate “stress erythropoiesis” by producing BMP4, which is required for expansion and differentiation of erythrocyte progenitors to EPO-responsive BFU-E [7].

**Figure 4:**
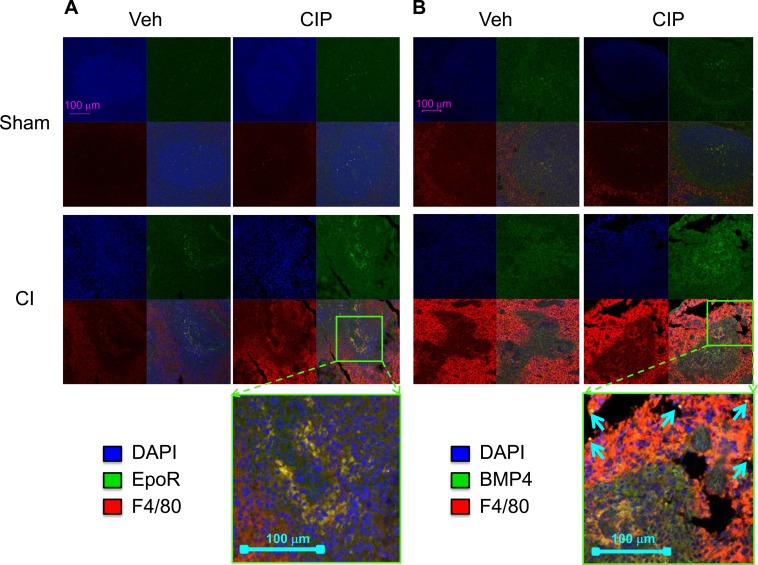
CIP stimulates BMP4 expression on F4/80^+^ cells after CI. Mice received CI or Sham and were treated with daily, oral doses of CIP or Veh starting 2 hours after injury. Spleens isolated from mice 10 days after injury were used for immunofluorescent staining followed by microimaging analysis. For both (A) and (B), Top left: Sham-Veh; Top right: Sham-CIP; Bottom left: CI-Veh; and Bottom right: CI-CIP. Annotation in top-left image applies to all other images unless otherwise noted. Staining for CI-CIP was magnified and presented at bottom of figure as indicated. (A) For each panel, upper left quadrant: DAPI; upper right quadrant: EpoR; lower left quadrant: F4/80; and lower right quadrant: overlay of other three quadrants. (B) For each panel, upper left quadrant: DAPI; upper right quadrant: BMP4; lower left quadrant: F4/80; and lower right quadrant: overlay of other three quadrants. CIP: ciprofloxacin; Veh: vehicle; CI: combined injury; Sham: free of any injury.

We wanted to identify splenocyte subsets that express EpoR, F4/80 and BMP4 at protein levels that are responsible for stress erythropoiesis. We, therefore, analyzed cellular distribution of EpoR and F4/80 (Figure 4A), as well as F4/80 and BMP4 (Figure 4B), by immunofluorescent double staining. In general, CI induced an increased distribution of F4/80^+^ cells, which dominated and clearly distinguished red pulp from white pulp (Figure 4A and B bottom panels). It has been reported that red pulp macrophages (RPMs) digest apoptotic cells, including erythrocytes [16]–[18]. In white pulp, there were many fewer though significant numbers of F4/80^+^ cells detected after CI, and EpoR was consistently present in this population (Figure 4A bottom panels). A magnified image of EpoR in the spleen of a representative CIP-treated CI mouse is presented at the bottom of Figure 4A. Very low levels of EpoR were detected in spleens of Sham mice (Figure 4A top panels). Significant levels of BMP4 were also detected on F4/80^+^ macrophages in white pulp that became more intense with CIP treatment (Figure 4B bottom panels). A magnified image of BMP4 in the spleen of a representative CIP-treated CI mouse is presented at the bottom of Figure 4B. Interestingly, very high and specific expression of BMP4 in F4/80^+^ cells was also observed in RPMs of CIP-treated CI mice (see arrows in Figure 4B, far bottom).

### Ciprofloxacin Primes Stress Burst-Forming Unit-Erythroid Formation after CI

**Figure 5:**
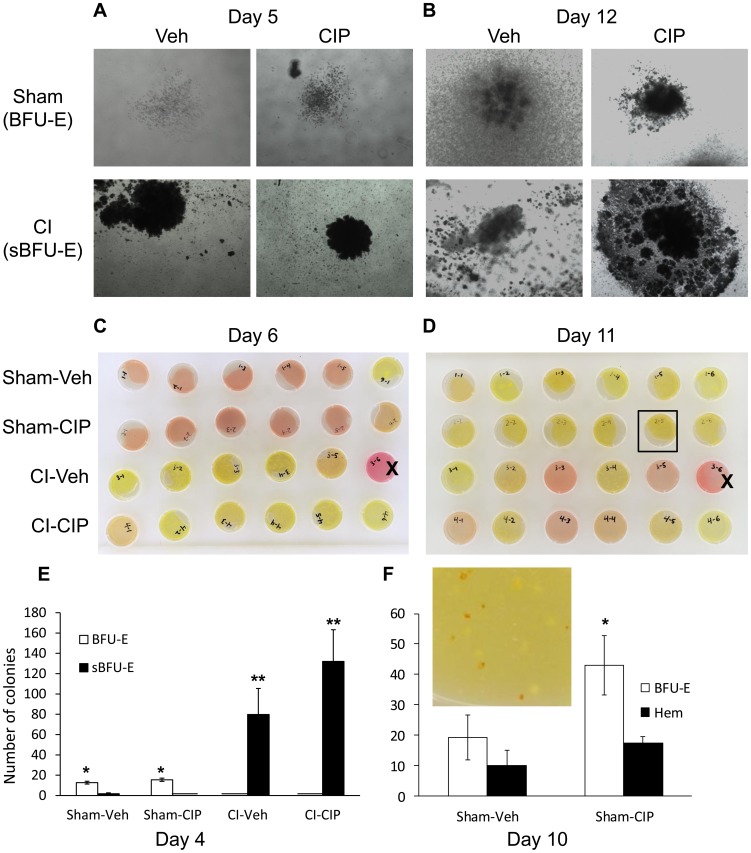
Enhanced ability of stress BFU-E formation from the splenocytes of CIP-treated CI mice. Mice received CI or Sham and were treated with daily, oral doses of CIP or Veh starting 2 hours after injury. Splenocytes were isolated and inoculated from mice 10 days after injury. (A and B) Representative images of BFU-E and stress BFU-E that formed in early stage (4–6 days after *ex vivo* inoculation; A) and late stage (10–12 days after *ex vivo* inoculation; B). (C and D) Images of dishes containing splenocytes in early (C) and late (D) stages after *ex vivo* inoculation. Boxed dish in (D) was magnified in (F) inset. “X” signifies dishes that demonstrated bacterial growth. (E) Numbers of BFU-E and stress BFU-E in the early stage (day 4). *p<0.01, Sham vs. CI BFU-E. **p<0.01, Sham vs. CI sBFU-E. There were no sBFU-E in Sham-CIP, and no BFU-E in CI-Veh and CI-CIP. (F) Numbers of BFU-E and hemoglobinized BFU-E in late stage (day 10). Sham-Veh and Sham-CIP were studied. *p<0.1 compared to BFU-E in other group. CIP: ciprofloxacin; Veh: vehicle; CI: combined injury; Sham: free of any injury; sBFU-E: stress BFU-E; Hem: hemoglobinized.

It was possible that splenocytes from CIP-treated CI mice gave rise to more efficient production of erythrocytes aided by the mechanisms described above. To assess this possibility, we isolated fresh splenocytes from mice 10 days after CI, and studied their ability to form stress burst-forming unit-erythroid (sBFU-E) in a conditioned medium *ex vivo*. We analyzed colonies formed during early (days 4–6) and late (days 10–12) stages. In the early stage, many colonies formed in the dishes with splenocytes from CI mice (sBFU-E), while very few formed from Sham mice (BFU-E). Colonies grown from CI-mouse splenocytes were morphologically different from those from Sham mice (Figure 5A top vs. bottom panels). We also noticed a significant color change in the medium with CI splenocytes during the early stage, which may be associated with greater cell growth rates (Figure 5C top 2 rows vs. bottom 2 rows). In the late stage though, we found colony formation in dishes with splenocytes from Sham mice with color change in medium (Figure 5D top 2 rows). Some of these colonies displayed bright red color indicating hemoglobinized erythrocytes being formed while others remained white (Figure 5F inset for magnification of boxed-plate in Figure 5D). These hemoglobinized cells were also morphologically distinct (Figure 5B top vs. bottom panels) and demonstrated that homeostatic erythropoiesis can take place in spleen as well as bone marrow. The number of colonies found in early and late stages was scored and presented (Figures 5E and F). By day 4, the splenocytes from CIP-treated CI animals had formed a greater number of sBFU-E than those from Veh-treated ones (Figure 5E marked sBFU-E), whereas the number of BFU-E in Sham groups showed no difference, with or without CIP treatment (Figure 5E marked BFU-E). We counted colonies grown from Sham-derived splenocytes at day 10 and found the number of BFU-E became significantly greater in CIP-treated than in Veh-treated specimens (Figure 5F, marked BFU-E). We did not see an increase in the number of newly hemoglobinized colonies with the CIP treatment at this point (Figure 5F, marked Hem). However, the growth rate of BFU-E observed in Sham-CIP splenocytes was significantly higher than that measured in Sham-Veh by day 10 (Figure 5E vs. F). CIP treatment may thus result in better formation of hemoglobinized cells after longer incubation. Inoculation of splenocytes from one of the CI-Veh animals (#3–6) resulted in growth of bacteria (the media contained no antimicrobial agents), suggesting this animal contained a septic spleen (marked with Xs in Figures 5C and D).

### Ciprofloxacin Promotes Stress Erythropoiesis under CI

**Figure 6:**
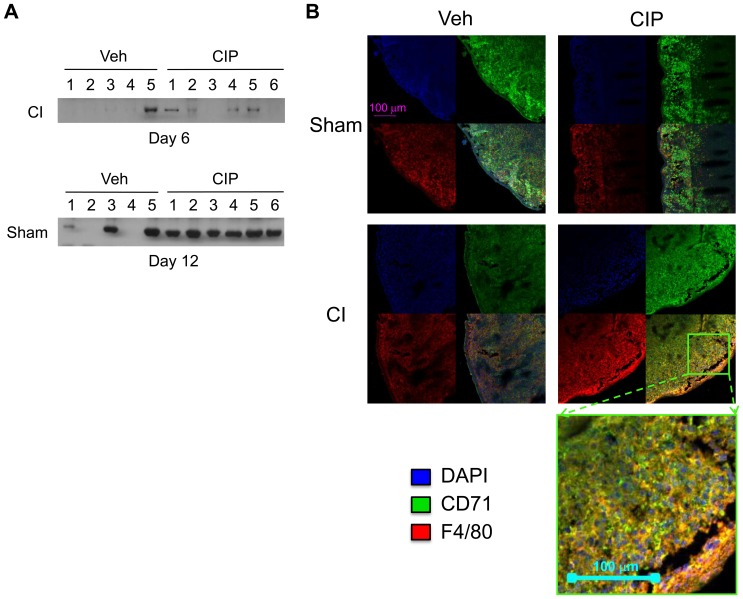
CIP enhances stress erythropoiesis after CI. Mice received CI or Sham and were treated with daily, oral doses of CIP or Veh starting 2 hours after injury. Spleens isolated from mice 10 days after injury were used for splenocyte inoculation followed by immunoblot analysis (A) or immunofluorescent staining followed by microimaging analysis (B). (A) Presence of CD71, a CFU-E marker, in colonies harvested in early (top) and late (bottom) stages. Insufficient number of splenocyte colonies prevented analysis of Sham at day 6; analysis was successful at day 12. (B) Top left: Sham-Veh; top right: Sham-CIP; bottom left: CI-Veh; and bottom right: CI-CIP. In each panel, upper left quadrant: DAPI; upper right quadrant: CD71; lower left quadrant: F4/80; and lower right quadrant: overlay of other three quadrants. Annotation in top-left image applies to all other images unless otherwise noted. Staining for CI-CIP was magnified and presented at bottom of figure as indicated. CIP: ciprofloxacin; Veh: vehicle; CI: combined injury; Sham: free of any injury.

To examine whether the *ex vivo* growing cells described above contain CFU-E, immunoblot analysis was performed on the cell lysates obtained from the colonies harvested on days 6 and 12. Antibodies to CD71 (marker for CFU-E) and Ter-119 (a late marker for erythrocyte lineage) were used. In day 6 samples, we analyzed colonies only from CI animals, as we did not obtain enough colonies in Sham samples at this time (Figure 6A). We did not find significant expression of Ter 119, as would be expected at this early time point (data not shown). However, expression of CD71was detected in one of five Veh-treated and four of six CIP-treated specimens (Figure 6A top panel). In the later time point at day 12, we found significant formation of analyzable colonies from splenocytes of Sham mice. Expression of CD71was significant in two of five Veh-treated and six of six CIP-treated specimens, indicating homeostatic erythropoiesis was more active after CIP treatment (Figure 6A bottom panel).

Finally, we studied the distribution of CD71 in the spleens of CI and Sham-treated mice with or without CIP treatment. In Sham mice, there were significant numbers of CD71^+^ erythroblasts that surrounded F4/80^+^ macrophages regardless of CIP treatment (Figure 6B top panels). This microenvironment has been described as erythroblastic islands: niches for erythropoiesis [19]. After CI, there was significant loss of CD71 expression but CIP restored it, suggesting CIP supports stress erythropoiesis (Figure 6B bottom panels). A magnified image for CD71 in the spleen of a representative CIP-treated CI animal is presented at the bottom of Figure 6B.

## Discussion

Previous research has indicated that prevention of endogenous bacterial translocation via the intestinal barrier and subsequent sepsis in multiple organs is an indispensable component of CI treatment [1]. We have used CIP and several other FQs in previous studies to accomplish this goal [unpublished data].

To date, CIP has been approved by the FDA for bacterial infection control. Many animal experiments and clinical studies have also revealed immunomodulatory effects of FQs in various disease and injury models [20]. Such studies include several relevant to the radiation countermeasure field. For example, CIP in combination with bone marrow transplantation (BMT) was shown to increase 20-day survival of lethally irradiated mice better than BMT alone and is associated with enhanced hematopoiesis and peritoneal neutrophil function [21]. Also, various FQs were compared for their efficacies in the treatment of sub-lethally irradiated mice, demonstrating that three of the tested FQs, including CIP, enhanced hematopoiesis better than the others, although they did not lead to better survival [22].

CI in our model, as previously described, exhibits significantly different pathology than does RI [1]. Despite knowing that CIP does not greatly improve survival after RI, we pursued testing of this drug because several favorable pathophysiological modulations occurred in CIP-treated CI animals: cytokine and chemokine release in sera, bone marrow repopulation, and limitation of apoptosis and autophagy in ileum [3]. These multiple effects in different tissues – although lacking systematic explanation – suggested the benefit of using CIP for CI treatment.

We provide here the first evidence that CIP treatment significantly improves 30-day survival of CI mice, an observation that supports further development of CIP as a CI countermeasure. We also found CIP treatment did not significantly reduce mortality in RI animals, which validates observations by others using different strains of mice [22].

CIP-mediated immunomodulation in the CI model includes increased levels of IL-3 and decreased levels of several proinflammatory cytokines such as IL-6 in sera. IL-3 is known to stimulate differentiation of hematopoietic stem cells (HSCs) into myeloid lineages. However, we did not find any improvement in total number of peripheral white blood cells and platelets except that cellularity in bone marrow improved [3]. It is possible that the effect of CIP on hematopoiesis becomes more prominent under less severe CI conditions, or CIP may have enhanced alternate mechanisms developed under stress conditions, such as stress erythropoiesis.

The present study found that CIP treatment after CI significantly improved erythrocyte counts associated with better hemoglobin and hematocrit levels. Based on *ex vivo* studies, a previous investigation found no effect of CIP on erythropoiesis [23]. Erythropoiesis belongs to the myeloid lineage and requires IL-3 in combination with other cytokines including stem cell factor (SCF) and EPO [24]. Another report showed that inflammation mediated by IL-6 impairs iron availability, leading to iron-deficiency anemia [25].

Two independent mechanisms for erythropoiesis have been identified: homeostatic erythropoiesis and stress erythropoiesis. Homeostatic erythropoiesis occurs mostly in bone marrow and functions to maintain normal erythrocyte levels. CIP stimulated homeostatic erythropoiesis as seen in erythrocyte counts in Sham animals by day 3 (Figure 2B). EPO mRNA in kidney was also found to be significantly higher in Sham-CIP animals than in Sham-Veh animals on day 10 (Figure 3B, C), suggesting EPO induction may have played a role in stimulating the increase in erythrocytes. The transient increase in erythrocytes by CIP was also observed in CI animals at an early time point, such as day 1 (Figure 2D), suggesting CIP stimulated erythroid progenitors in bone marrow before they underwent radiation-induced apoptosis.

Stress erythropoiesis, on the other hand, occurs in spleen in response to anemic conditions. In the case of CI-associated pathology, severe anemia as well as loss in bone marrow cellularity triggers stress erythropoiesis, which requires BMP4 and the formation of stress BFU-E driven by EPO stimulation. While EPO is essential for the types of erythropoiesis discussed above, production of EPO in the kidney and elevated circulating levels were found to be high after CI regardless of the CIP treatment (Figure 3). Therefore, CIP-mediated EPO modulation does not appear to be a major determinant for enhanced erythropoiesis under CI condition. The data suggest CIP augmented stress erythropoiesis by a mechanism other than EPO induction, resulting in higher erythrocyte counts by day 10.

Our histopathological study of CI spleens indicated a co-localization of EpoR and F4/80^+^ macrophages, which at the same time produced BMP4 essential for stress erythropoiesis (Figure 4). It has been reported that macrophages in general are markedly radio-resistant compared to other hematopoietic populations [26], and RPMs are crucial for erythroid recovery after myeloablation [27], [28]. Since CIP increased specific BMP4 distribution in those populations, it became necessary to observe their ability to form BFU-E. Indeed, our observation revealed enhanced formation of sBFU-E in splenocytes isolated from CIP-treated CI animals relative to those of Veh-treated CI animals (Figure 5). CIP must have stimulated splenocytes and their ability to form BFU-E prior to isolation of spleen since CIP was not added to the *ex vivo* cultures. *Ex vivo* colonies and spleens of CIP-treated CI animals consistently exhibited marked restoration of CD71 distribution surrounding an F4/80^+^ population, presumably providing a niche critical for erythropoiesis [27].

Interestingly, we have shown that CIP treatment after CI is even more effective at improving 30-day survival than the combination therapy of orally administered levofloxacin (LVX) and topical gentamicin application on wounds [unpublished data]. The fact that LVX is one of the FQs with a broader spectrum of antimicrobial effects than CIP indicates that controlling bacterial infection alone is not sufficient for CI treatment and that CIP employs other mechanisms of action not seen with LVX or gentamicin. Thus, our research has shown not only an efficacy of CIP superior to other FQs tested, but has also identified key mechanisms of action that could help advance medical management of CI.

The CIP regimen used in this study was originally optimized for severe sepsis seen after CI. This regimen uses higher but fewer doses of CIP than other studies [22]. It is well-tolerated and may prove to be a safer application of this widely-used drug to lessen the development of bacterial resistance [29]. The study even opens the possibility that the 3-week regimen we used could be shortened, as no further mortality was observed upon its completion. It will be useful to determine if this superior regimen could treat more severe CI, even when much higher doses of radiation combined with wounding would lead to no expectation of survival.

In summary, CIP treatment 1) significantly improved survival after CI; 2) partially ameliorated CI-induced severe anemia; 3) increased BMP4 production by F4/80^+^ macrophages in spleen; and 4) elevated IL-3 concentrations in serum of CI mice. *Ex vivo* splenocytes isolated from CIP-treated CI mice formed more CFU-E in the presence of IL-3 and EPO, which are elevated after CI. These results suggest that CIP effectively mitigates CI-induced severe anemia and mortality.

## Materials and Methods

### Ethics Statement

Research was conducted in a facility accredited by the Association for Assessment and Accreditation of Laboratory Animal Care-International (AAALACI). All procedures involving animals were reviewed and approved by the AFRRI Institutional Animal Care and Use Committee. Euthanasia was carried out in accordance with the recommendations and guidelines of the American Veterinary Medical Association. For the survival study, we observed animals every 2 hours during work hours, and moribund animals were euthanized according to humane endpoints. The clinical definition of moribund is being in the state of dying with no expectation of recovery, where animals display a combination of the following: lowered body temperature, slow or impaired motion, continuous shaking, hunched back, and inability to maintain sternal recumbency. Moribund animals were placed in a separate cage where carbon dioxide gas was applied until no breathing was observed, followed by a cervical dislocation as a secondary confirmatory method of euthanasia. In some cases, we confirmed animals that have died early in the morning. Deceased animals were immediately removed from the cages to avoid any health problems caused by means other than the experimental treatment. Any surviving animals at the end of the study were subjected to euthanasia, also by the application of carbon dioxide followed by cervical dislocation. For the studies other than those testing survival, mice at specific endpoints were placed under anesthesia by isoflurane inhalation for the entire period of blood collection, immediately followed by a confirmatory cervical dislocation for euthanasia and terminal tissue collection. All efforts were made to minimize suffering.

### Animals

Female B6D2F1/J mice were purchased from Jackson Laboratory (Bar Harbor, ME) and were used between 12 to 20 weeks of age. Male mice were not used in this study because of problems associated with aggression, which in these experiments could lead to further damage to wound sites and enhanced infection. All mice were randomly assigned to experimental groups. No more than 4 mice were housed per filter-topped polycarbonate MicroIsolator (Allentown Caging, Allentown, NJ) in conventional holding rooms. Rooms were provided with 10–15 changes per hour of 100% fresh air conditioned to 72±2°F with a relative humidity of 50±20%. Mice were maintained on a 12-hour light/dark, full-spectrum light cycle with no twilight.

A few days prior to experiments, mice were weighed and electric clippers were used to remove the hair of the dorsal surface under anesthesia (methoxyflurane or isoflurane inhalation). On the day of experiments, mice were first irradiated and then wounded within 1 hour of the time of irradiation completion. All mice, including controls, received an intraperitoneal injection of 0.5 mL sterile isotonic 0.9% NaCl as fluid therapy immediately after combined injury to avoid radiation-induced dehydration. After CI, mice were reassigned to clean cages and provided with proper food, acidified water, cotton for nesting and a plastic dome.

### Irradiation

Mice were placed in well-ventilated acrylic restrainers and given specified doses of whole-body ^60^Co γ-photon irradiation delivered at a dose rate of approximately 0.4 Gy/min. Dosimetry was performed using the alanine/electron paramagnetic resonance system. Calibration of the dose rate with alanine was directly traceable to the National Institute of Standards and Technology and the National Physics Laboratory of the United Kingdom. Sham-irradiated mice were placed in acrylic restrainers, taken to the radiation facility, and held there for the time required for irradiation without experiencing radiation exposure.

### Wound Trauma

After irradiation, mice were anesthetized by methoxyflurane or isoflurane inhalation prior to wounding. In the case of isoflurane use, animals received acetaminophen immediately after wounding (150 mg/kg, *i.p.*). An experimental wound was administered 19±1.3 mm from the occipital bone and between the scapulae using a stainless steel punch on a Teflon®-covered board cleaned with 70% alcohol before each use. The panniculus carnosus muscle and overlying skin (23.5±1.1 mm long and 14.9±0.7 mm wide) were removed. Sham-wounded mice were treated identically to other groups except without wounding.

### Preparation and Administration of CIP

Veterinary-, oral-use CIP tablets (500 mg/each) (Dr. Reddy’s Laboratories, Hyderabad, India) were used to prepare a fresh solution each week. Tablets were ground, dissolved in sterile water (vehicle) and after brief centrifugation sterile-filtered using a.22 µm cellulose nitrate (CN) filter system (Corning, Corning, NY). Each dose of 0.2 mL CIP solution was calculated to deliver 90 mg/kg of CIP, based on the average mouse weight taken prior to the experiment (see “*Animals*”). Mice were orally administered 0.2 mL of either CIP or vehicle once daily for 3 weeks, starting within 2 h of combined injury (day 0) and ending with the 22^nd^ dose (day 21) unless otherwise noted. Mice were gently restrained by hand and fed using oral feeding needles attached to 1 mL syringes. Feeding needles were sterilized between each use by 70% ethanol and new needles were used for every cage.

### Wound Closure

Assessments of wound closure were performed on days 1, 7, 14, 21 and 28. Wounds were measured to within 0.01 mm by a caliper with an electronic digital display. The average area of each wound was calculated as π (diameter A/2) x (diameter B/2), where A and B represent diameters at right angles to each other.

### Histopathology Assessment, Immunofluorencent Staining and Confocal Microscopic Analyses

Spleen and kidney specimens were collected from euthanized animals on the days specified in each figure. Specimens were immediately fixed in 10% phosphate-buffered formalin upon removal, and then embedded in paraffin, sectioned transversely and stained with hematoxylin and eosin (H & E). Tissue imaging and analysis were performed with the NanoZoomer 2.0 from Hamamatsu Photonics K.K. (Hamamatsu, Japan). In some experiments, unstained paraffin sections were used for immunofluorescent staining. Paraffin sections on slides were treated with Target Retrieval Solution and Protein Block Serum-Free (Dako North America, Inc., Carpinteria, CA) according to the manufacturer’s protocol, and stained with respective primary and secondary antibodies with washing between and after in phosphate-buffered saline (PBS) with 0.1% Tween® 20. Resulting slides were briefly rinsed with PBS, desalted by dipping in distilled-deionized water, and sealed with coverslips in mounting medium with DAPI (Life Technologies Corporation, Grand Island, NY). A Zeiss LSM710 laser scanning confocal microscope (Carl Zeiss MicroImaging; Thornwood, NY) with EC Plan-Neofluar 10x/0.3, Plan-Apochromat 20x/0.8, and EC Plan-Neofluar 40x/0.75 objectives was used to scan the signals. Intensities of signals were also measured and shown as noted.

### RNA Extraction and Real-Time Quantitative Reverse Transcription-PCR (qRT-PCR)

Total RNA was extracted using RNeasy (Qiagen, Valencia, CA). Synthesis of cDNAs and subsequent PCR reactions were performed using the ThermoScript™ RT-PCR System plus Platinum® Taq DNA Polymerase (Life Technologies). Real-time PCR reactions were performed using iQ™ SYBR® Green Supermix (Bio-Rad, Hercules, CA) following the manufacturer’s protocol. As an internal control, each amplification rate of a target gene was normalized by the one obtained from the hypoxanthine-guanine phosphoribosyltransferase (HPRT) gene using the same cDNA template.

### Tissue Preparation for Immunoblots

Kidney and spleen specimens were collected from euthanized animals on the days specified in each figure. Tissues were minced, mixed with lysis buffer (see “*Reagents*”), sonicated for 15 sec on ice, and then centrifuged at 10,000 g for 10 min at 4°C. Supernatants were conserved for determination of protein contents and immunoblot analysis. Protein contents in cell lysates were determined by Bio-Rad Protein Assay (Bio-Rad, Richmond, CA). Total cell lysates were boiled in the presence of final concentrations of 1x LDS sample buffer (Life Technologies Corporation) with 10% β-mercaptoethanol (Life Technologies) for 5 min. Samples were briefly pelleted by centrifugation and kept on ice until separation by NuPAGE® 4–12% Bis-Tris gel in running buffer (Life Technologies Corporation). Separated proteins in gels were transferred to 0.45 µm pore-size PVDF membranes (Life Technologies Corporation) in 1x transfer buffer (Life Technologies). Membranes were then soaked in blocking buffer, which contained 3% non-fat dry milk (Santa Cruz Biotechnology, Santa Cruz, CA) dissolved in Tris-buffered saline (50 mM Tris-HCl, pH 8.0, and 150 mM NaCl) supplemented with 0.2% Tween® 20 (TBST). Blocked membranes were reacted with the primary antibody followed by the secondary antibody against specific antigens and washed with TBST after each reaction. Resulting membranes were reacted with ECL reagents (Amersham; Piscataway, NJ) and exposed to Kodak BioMax Light films (Kodak; Rochester, NY) to identify bands.

### EPO Measurement in Sera

Whole blood (0.7–1 mL) was collected in CapiJect tubes (Terumo, Somerset, NJ) by cardiac puncture from mice anesthetized with Isoflurane on the days specified in each figure. Sera were separated by centrifugation at 3,500 g for 90 sec and stored at −80°C until assayed. Serum from each animal was examined in duplicate. The Mouse/Rat Erythropoietin Quantikine ELISA Kit (R&D Systems, Inc. Minneapolis, MN) was used according to the manufacturer’s instructions.

### Colony Formation Assay

Freshly isolated spleens from euthanized animals were used. They were first minced by sterile scissors and then gently mashed on a nylon mesh cell strainer with pore size of 40 µm to collect splenocyte single cell suspensions (StemCell Technologies Inc., Vancouver, Canada). Collected cells were washed, counted by a C-Chip Hemocytometer (Cheonan, Korea), and adjusted to 2×10^6^ cells/mL. The same numbers of cells were seeded in MethoCult® media according to the manufacturer’s instruction (StemCell Technologies Inc.).

### Reagents

Lysis buffer contained 20 mM HEPES (pH7.2–7.5) (Life Technologies), 150 mM NaCl (Sigma-Aldrich; St. Louis, MO), 0.5% Nonidet P40 (Roche; Indianapolis, IN), in the presence of protease inhibitors and phosphotase inhibitors (Sigma-Aldrich, St. Louis, MO). Antibodies specific to the following antigens were used: β-Actin (Sigma-Aldrich); HIF-1α, EpoR and rabbit or mouse IgG with hrp conjugations (Santa Cruz Biotechnology, Inc. Santa Cruz, CA); F4/80 and BMP4 (abcam® Cambridge, MA); Transferrin Receptor, Alexa Fluor® 488 goat anti-rabbit IgG (H+L), Alexa Fluor® 568 goat anti-rat IgG, and Alexa Fluor® 647 goat anti-rabbit IgG (H+L) from Life Technologies. Primer sets for PCR amplification were as follows: mEPO: Forward 5′- TCT TAG AGG CCA AGG AGG CAG AAA-3′ and Reverse 5′- ACC CGG AAG AGC TTG CAG AAA GTA-3′ (385 bp); and mHPRT: Forward 5′- TCT CGA AGT GTT GGA TAC AGG CCA-3′ and Reverse 5′- AGC TTT ACT AGG CAG ATG GCC ACA-3′ (253 bp).

### Statistical Analysis

For survival experiments, ten mice were used for each group, and the experiment was repeated (*n* = 20/group). Survival plots are shown with significance analyzed by Probit statistics (the Mantel-Cox test). The intensities of immunoblot and PCR signals were quantified by the Gel Logic 2200 PRO Imaging System (Carestream Health, Inc. Woodbridge, CT). All statistical results are expressed as means ± SEM, and *n* is provided in figure legends when necessary. One-way ANOVA, two-way ANOVA, Studentized-range test, and the Chi^2^ test were used for comparison of groups; 5% was used as the level of significance unless otherwise noted.
